# Competing endogenous RNA networks and ferroptosis in cancer: novel therapeutic targets

**DOI:** 10.1038/s41419-024-06732-4

**Published:** 2024-05-22

**Authors:** Fatemeh Nejadi Orang, Mahdi Abdoli Shadbad

**Affiliations:** 1 Immunology Research Center, Tabriz University of Medical Sciences, Tabriz, Iran; 2 Student Research Committee, Tabriz University of Medical Sciences, Tabriz, Iran; 3 Department of Immunology, Tabriz University of Medical Sciences, Tabriz, Iran

**Keywords:** Cell signalling, Cancer

## Abstract

As a newly identified regulated cell death, ferroptosis is a metabolically driven process that relies on iron and is associated with polyunsaturated fatty acyl peroxidation, elevated levels of reactive oxygen species (ROS), and mitochondrial damage. This distinct regulated cell death is dysregulated in various cancers; activating ferroptosis in malignant cells increases cancer immunotherapy and chemoradiotherapy responses across different malignancies. Over the last decade, accumulating research has provided evidence of cross-talk between non-coding RNAs (ncRNAs) and competing endogenous RNA (ceRNA) networks and highlighted their significance in developing and progressing malignancies. Aside from pharmaceutical agents to regulate ferroptosis, recent studies have shed light on the potential of restoring dysregulated ferroptosis-related ceRNA networks in cancer treatment. The present study provides a comprehensive and up-to-date review of the ferroptosis significance, ferroptosis pathways, the role of ferroptosis in cancer immunotherapy and chemoradiotherapy, ceRNA biogenesis, and ferroptosis-regulating ceRNA networks in different cancers. The provided insights can offer the authorship with state-of-the-art findings and future perspectives regarding the ferroptosis and ferroptosis-related ceRNA networks and their implication in the treatment and determining the prognosis of affected patients.

## Facts

- Ferroptosis is a newly identified regulated cell death that has anti-tumoral properties.
- Restoring ferroptosis enhances cancer immunotherapy and chemoradiotherapy responses.
- The dysregulation of lncRNA and circRNA-mediated ceRNA networks is highly implicated in tumor development and progression.
- Restoring dysregulated ferroptosis-related ceRNAs not only stimulates ferroptosis but also decreases tumor growth and oncogenesis and increases anti-neoplastic treatment efficacy.

## Open questions

- What are the regulated cell deaths, and what is ferroptosis?
- What are the signaling pathways of ferroptosis?
- What is the significance of ferroptosis in oncogenesis and anti-neoplastic treatments?
- What is the concept behind the competing endogenous RNA networks?
- Can regulating ferroptosis-related competing endogenous RNA networks be a therapeutic option for cancer treatment?

## Introduction

Solid cancers are a prominent contributor to cancer-related mortality, as identified by tumor recurrence and metastasis [1]. The development and progression of solid cancers is a complex process influenced by genetic mutations within cancer cells and the surrounding microenvironment [2].

The significance of ncRNA in cellular physiology is well-established. It has been reported that *organims like worm and plant* have approximately comparable protein-coding genes with humans [3]; however, the extant and magnitude of ncRNA is much larger than these lower organisms [4]. This highlights the significance of ncRNA in maintaining homeostasis and developing higher eukaryotes. Consistent with this, accumulating evidence has shed light on the importance of dysregulated ncRNA expression profiles in developing various solid cancers [5]. Also, restoring and modulating the expression of ncRNAs have shown promising results in treating various malignancies in pre-clinical studies [6]. In the era of targeted therapies, the complex interactions between miRNA, lncRNA, and circRNA and the concept of ceRNA networks have opened in new chapter in studying various aspects of oncogenesis and developing novel treatments.

Cell death is a physiological process that occurs during the development of multicellular organisms. Accidental cell death (ACD) and regulated cell death (RCD) are two types of cell death that differ in their underlying causes and mechanisms [7]. ACD is a type of cell death that occurs due to external factors that cause physical or chemical damage to the cell. On the other hand, RCD is a controlled form of cell death that occurs through internal signaling pathways and is tightly regulated. Apoptosis, autophagy, necroptosis, pyroptosis, parthanatosis, ferroptosis, and cuproptosis are among the regulated cell deaths, each with distinct molecular mechanisms and characteristics [7–9].

The present study aimed to review the biology of miRNA, lncRNA, circRNA, and ceRNA concepts along with ferroptosis, ferroptosis pathways, the significance of ferroptosis in cancer and anti-neoplastic treatments. Furthermore, the current review provides the authorship with the current evidence on the ferroptosis-regulating ceRNA networks in solid cancers and sheds light on the novel approach to regulating tumor ferroptosis, i.e., thorough ferroptosis-regulating ceRNA networks.

## Non-coding RNAs and ceRNA networks

Approximately 80% of the human genome is transcribed into RNAs that do not code for proteins; these ncRNAs have critical regulatory functions in the initiation and progression of different types of cancer [10]. NcRNAs can be categorized into various classes based on their size and function. Among them, microRNAs (miRNAs), long non-coding RNAs (lncRNAs), and circular RNAs (circRNAs) have been demonstrated to play significant roles in ferroptosis, thereby impacting tumor growth [11].

MiRNAs are a class of small non-coding RNA molecules that regulate gene expression by binding to complementary sites on targeted mRNA. The biogenesis of miRNAs in humans follows a two-step process involving nuclear and cytoplasmic cleavage events [12]. MiRNA genes are first transcribed by RNA polymerase II in the nucleus, resulting in primary miRNA transcripts (pri-miRNAs) that contain stem-loop structures [13]. The microprocessor complex, consisting of the RNase III enzyme Drosha and its cofactor DGCR8, identifies and cleaves these stem-loop structures to release hairpin-shaped precursor miRNAs (pre-miRNAs) [14]. Pre-miRNA is transported into the cytoplasm by interacting with RanGTP/Exportin-5 [15]. In the cytoplasm, the pre-miRNA undergoes cleavage mediated by the RNase III enzyme Dicer-1, together with TRBP/PACT proteins [16, 17]. This cleavage process results in a short double-stranded miRNA comprising both a guide and passenger strand. Once these two miRNA strands are separated, the guide strand is loaded into the RNA-induced silencing complex (RISC) to form the miRISC, which includes Dicer1 and Argonaute proteins. The miRNA guides RISC to identify complementary sequences on target mRNAs, resulting in the repression of gene expression [17, 18]. In light of their significant regulatory roles, it is apparent that modifications in miRNA expression have been associated with human diseases, including various cancers [17].

LncRNAs are transcripts that lack the protein-coding ability and possess a minimum length of 200 nucleotides [19]. They play essential roles in a wide range of physiological and pathological processes. Aberrant lncRNA expression levels have been observed in various cancers [10]. lncRNAs can undergo a variety of processing mechanisms, such as cleavage by ribonuclease P (RNase P) to create mature 3’ ends, capping by small nucleolar RNA (snoRNA) and proteins (snoRNP) complexes at their ends, or the formation of circular structures that protect them from degradation [19]. In contrast to mRNAs, many lncRNAs transcribed by RNA polymerase II remain in the nucleus due to inefficient processing, while others are spliced and exported to the cytoplasm [20]. The cytoplasmic export of lncRNAs (and mRNAs) that contain one or a few exons is mediated by nuclear RNA export factor 1 (NXF1) [21]. In the cytoplasm, lncRNAs can undergo specific sorting processes that assign different lncRNAs to specific organelles (e.g., mitochondria, exosomes) or be distributed in the cytoplasm and interact with diverse RNA-binding proteins [20]. Based on their genomic localization, lncRNAs are divided into sense, anti-sense, bidirectional, intronic, and intergenic lncRNAs [22].

CircRNAs are single-stranded RNA transcripts derived from a noncanonical type of alternative splicing known as back-splicing. Unlike linear RNA, circRNA creates a covalently closed loop structure lacking 5′ and 3′ ends [23]. This closed-loop structure renders circRNAs less susceptible to exonuclease degradation [24]. CircRNAs can be categorized into four distinct groups: exonic circRNAs (EcRNAs), exon-intron circRNAs (EIciRNAs), circular intronic RNAs (ciRNAs), and tRNA intronic circular RNAs (TricRNAs). Back-splicing in most circRNAs involves the spliceosome machinery joining the downstream 5′ splice-donor site to the upstream 3′ splice-acceptor site, creating a closed-loop structure with a specific junction site. The process is regulated by both cis-acting elements and trans-acting splicing factors [25]. Cis-acting regulatory elements that contain reverse complementary sequences (e.g., Alu repeats) flanking an intron through complementary pairing drive exon cyclization to form EIciRNAs and EcRNAs [26]. Trans-acting factors, such as RNA-binding proteins (e.g., QKI, MBL), can identify and attach to specific sites on the pre-mRNA flanking introns, forming EIciRNAs and EcRNAs [25, 26]. Another mechanism for circRNA biogenesis is lariat-driven circularization, which occurs during exon-skipping events or intron removal from pre-mRNAs. In exon-skipping events, the skipped exonic and intronic sequences are excised from pre-mRNA and form a loop-like structure called a lariat. The lariat further undergoes internal splicing to eliminate intronic sequences, producing an EcRNA [27]. During intron removal from pre-mRNA, the processing of the intronic lariat relies on a motif that comprises a seven nucleotide GU-rich element near the 5′ splice site and an 11 nucleotide C-rich element close to the branchpoint site, and these sites are resistant to degradation by de-branching enzymes, resulting in the formation of circRNA [28]. Most circRNAs originate from pre-mRNA, whereas a minor part of intron-derived circRNAs is from pre-tRNA. In the process of pre-tRNA maturation, the cleavage of an intron-containing pre-tRNA can occur through the tRNA splicing endonuclease (TSEN) complex at the bulge-helix-bulge (BHB) motif. Then, the enzyme RtcB ligase joins the resultant intron termini, forming a stable circRNA known as TricRNAs [29]. The aberrant regulation of circRNAs has been implicated in developing various diseases like cancers [30].

In 2011, Salmena et al. described a hidden language between RNAs mediated by miRNA response elements [31]; this hypothesis was driven by their findings showing that pseudogenes could compete with ancestral protein-coding genes to bind with miRNAs [32]. As ncRNAs, circRNAs and lncRNAs possess miRNA response elements that lead to establishing a dynamic competition with mRNAs. If the expression of circRNAs and lncRNAs are upregulated, they bind to miRNAs, which leads to the liberation of mRNA expression. However, If the expression levels of circRNAs and lncRNAs are downregulated, mRNA binds to the miRNAs, which results in mRNA degradation [33]. This hypothesis provides logical justification for the lncRNA/miR/mRNA and lncRNA/miR/mRNA axes [34]. This ceRNA hypothesis also provides novel insights into another side of mRNA, i.e., its non-coding function in regulating other RNAs [31]. It is worth mentioning that although miRNAs are the central point of this competition, RNA-biding proteins can also regulate miRNAs, adding to the complexity of RNA regulation [35].

## Regulated cell death and ferroptosis

As a regulated cell death, pyroptosis is initiated by activating multiprotein complexes known as inflammasomes and subsequent activation of inflammatory caspases, such as caspase 1/4/5 in humans, leading to the cleavage of the N-terminal of gasdermin D (GSDMD) [36, 37]. Necroptosis is characterized by the activation of receptor-interacting protein kinase 1 (RIPK1; also known as RIP1), receptor-interacting protein kinase 3 (RIPK3; also known as RIP3), and their substrate is mixed-lineage kinase domain-like protein (MLKL), which facilitates its oligomerization and activation [38]. Parthanatos is a caspase-independent cell death initiated by over-activated poly (ADP-ribose) polymerase1 (PARP1) and the production of poly (ADP-ribose) (PAR) polymers. PAR polymers promote the translocation of apoptosis-inducing factor (AIF) from the mitochondria to the nucleus, which causes DNA degradation and ultimately results in cell death [7, 39]. Autophagy-dependent cell death is a type of RCD mediated by the molecular machinery of autophagy [40]. Cuproptosis occurs through the binding of copper to lipoylated components in the tricarboxylic acid (TCA) cycle, leading to the aggregation of lipoylated proteins and subsequent reduction in Fe–S cluster proteins, which induces proteotoxic stress and, eventually, cell death [8, 41].

Ferroptosis is a newly identified RCD characterized by the oxidation of polyunsaturated fatty acids (PUFAs) and the accumulation of lipid peroxides. The term ferroptosis was first coined in 2012 by Dr. Brent R. Stockwell’s lab [42]. According to their original study, comparing this iron-dependent cell death to other forms of RCD reveals distinct morphological, biochemical, and genetic differences [43]. The morphology of ferroptotic cells is characterized by smaller mitochondria, increased mitochondrial membrane density, decreased or absent cristae, outer mitochondrial membrane rupture, and the nuclei are normal in size without chromatin aggregation [44]. Accumulating studies have demonstrated that ferroptosis plays a crucial role in the development and progression of various diseases, including neurological disorders [45], cardiovascular diseases [46], ischemia-reperfusion injury [47], and cancer [48]. Regulation of ferroptosis primarily relies on the balance between glutathione (GSH) and ROS [49]. Ferroptosis inducers can be classified into two main groups. The first group, i.e., class I ferroptosis inducers, includes erastin, sorafenib, sulfasalazine, and glutamate, which function as system xc- blockers, causing a decrease in GSH levels. The second group, i.e., class II ferroptosis inducers, includes RAS synthetic lethal 3 (RSL3), ferroptosis-inducing agent 56 (FIN56), ferroptosis-inducing peroxide compound (FINO2), and ML162 (also known as DPI7), which inhibit the activity of glutathione peroxidase 4 (GPX4) [49–51]. Table 1 summarizes the morphological features, biochemical features, regulators, and inhibitors of well-known regulated cell deaths.

**Table 1:** Regulated cell death types.

Type	Morphological features	Biochemical features	Regulators	Inhibitors	References
Apoptosis	Cell shrinkage, pyknosis, membrane blebbing, condensation of chromatin, nuclear fragmentation	Phosphatidylserine exposure, activation of caspases, DNA breakdown	p53, Bax, Bak, Bcl-2, Bcl-XL	Z-VAD-FMK, Q-VD-OPh, Emricasan (IDN-6556)	[7, 151]
Pyroptosis	Cell swelling, nuclear condensation, formation of pores on the cell membrane, plasma-membrane rupture	Activation of inflammatory caspases, cleavage of Gasdermin D, Pro-inflammatory cytokine release	CASP1, CASP4, CASP5, GSDMD	MCC950, Glyburide, OLT1177, C7-09, Boc-D-FMK, Ac-YVAD-cmk, Ac-FLTD-CMK, Oridonin	[37, 152]
Necroptosis	Cell swelling, plasma membrane rupture, the release of damage-associated molecular patterns	Necrosome formation, drop in ATP levels, MLKL oligomerization, Release of DAMPs,	RIPK1, RIPK3, MLKL	Necrostatin-1 (Nec-1), Necrosulfonamide (NSA), GSK872, HS-1371	[38, 153]
Ferroptosis	Smaller mitochondria, decreased or vanished mitochondria cristae, mitochondrial outer membrane rupture.	Lipid peroxidation, iron and ROS accumulation, GSH depletion,	GPX4, TFR1, SLC7A11, NRF2, NCOA4, P53, FSP1, ACSL4, LPCAT3	Deferoxamine, Deferiprone, Cyclipirox, Ferrostatin-1, Liproxstatin-1	[43]
Parthanatos	Chromatin condensation, loss of plasma membrane integrity, mitochondria depolarization, considerable DNA fragmentation	AIF translocation from mitochondria to the nucleus, PARP1 hyperactivation, excessive generation of ROS	PARP1, AIFM1, MIF	AG14361, Iniparib, BYK204165	[7, 39]
Autophagy	Autophagic vacuolization, lysosomal degradation, formation of the phagophore, autophagosome, and autolysosome,	Increased lysosomal activity,	ULK1, LC3, ATGs, Beclin1, mTOR	3-methyladenine, chloroquine, bafilomycin	[40]
Cuproptosis	Not well established	Copper accumulation elevates ROS levels and proteotoxic stress.	FDX1, LIAS, DLAT, SLC31A1,	ATP7A/B, Glutathione	[41]

*CASP1* Caspase-1, *CASP4* Caspase-4, *CASP5* Caspase-5, *GSDMD* Gasdermin-D, *RIPK1* Receptor-interacting protein kinases 1 *RIPk*3, Receptor-interacting protein kinase 3, *MLKL* Mixed-lineage kinase domain-like protein, *GPX4* Glutathione peroxidase 4, *TFR1* Transferrin receptor 1, *SLC7A11* Solute carrier family 7 member 11, *NRF2* Nuclear factor erythroid 2-related factor 2, *NCOA4* Nuclear receptor co-activator 4, *ACSL4* Acyl-CoA synthetase long-chain family member 4, *LPCAT3* Lysophosphatidylcholine acyltransferase 3, FSP1 ferroptosis suppressor protein, *AIF* apoptosis-inducing factor, *PARP1* poly (ADP-ribose) polymerase-1, *ROS* reactive oxygen species, *AIFM1* apoptosis-inducing factor mitochondria-associated1, *DAMPs* damage-associated molecular patterns, *GSH* glutathione, *ULK1* Unc-51-like autophagy-activating kinase 1, *ATGs* autophagy-related genes, *mTOR* mechanistic target of rapamycin, *LC3* Microtubule-associated protein 1A/1B-light chain 3, *FDX1* Ferredoxin 1, *LIAS* Lipoic Acid Synthetase, *DLAT* Dihydrolipoamide s-acetyltransferase.

## Ferroptosis pathways

**Fig. 1:**
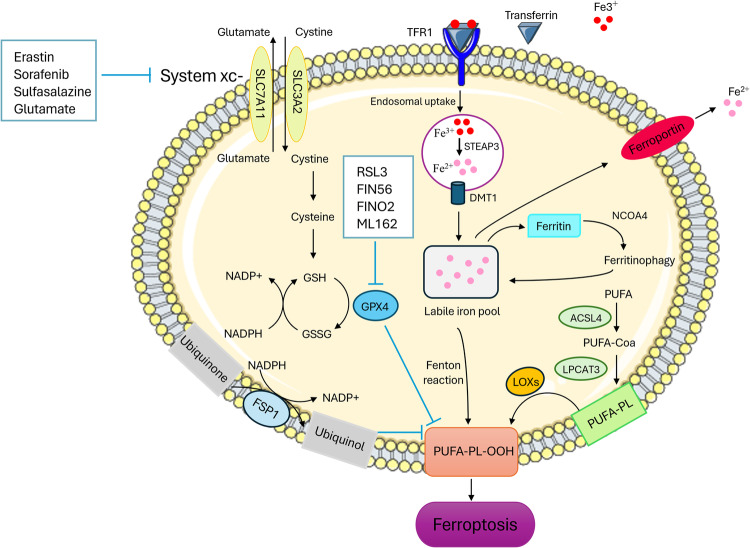
The signaling pathways of ferroptosis. The main metabolisms of ferroptosis can be categorized into three groups: iron metabolism, the GSH/GPX4 pathway, and lipid peroxidation. The System xc- antiporter mediates the exchange of intracellular glutamate and extracellular cystine. Cystine is converted to cysteine, a critical building block for GSH synthesis. GSH is an important substrate for the functioning of GPX4, which converts lipid hydroperoxides into their alcohol forms. GSH and GPX4 function as scavengers of reactive oxygen species to avert oxidative stress and lipid peroxidation. Another inhibitory mechanism of ferroptosis is regulated by FSP1–ubiquinol. Ferritinophagy, iron input, and output can employ the Fenton reaction to trigger lipid peroxidation and induce ferroptosis. The production and membrane deposition of PUFA-PLs requires the involvement of the enzymes ACSL4 and LPCAT3. SLC7A11, Solute carrier family 7 member 11; SLC3A2, Solute carrier family 3 member 2 ; TFR1, Transferrin receptor 1; RSL3, RAS synthetic lethal 3; FIN56, Ferroptosis-inducing agent 56; FINO2, Ferroptosis-inducing peroxide compound; GPX4, Glutathione Peroxidase 4; GSH, Glutathione; GSSH, glutathione counterpart; FSP1, Ferroptosis inhibitor protein 1; NADPH, Nicotinamide adenine dinucleotide phosphate; STEAP3, Six-transmembrane epithelial antigen of the prostate3; DMT1, Divalent metal transporter; NCOA4, Nuclear receptor co-activator 4; PFUA, polyunsaturated fatty acid; ACSL4, Acyl-CoA synthetase long-chain family member 4; LPCAT3, Lysophosphatidylcholine acyltransferase 3; LOX, Lipoxygenases; PUFA-PL, Polyunsaturated fatty acid phospholipids; PUFA-PL-OOH, Polyunsaturated fatty acid phospholipid hydroperoxides.

Ferroptosis regulation can be classified into two primary pathways. The first pathway involves metabolic processes associated with lipid, iron, and amino acid metabolism. The second pathway involves signaling pathways that regulate ferroptosis, including the tumor suppressor *TP53*, nuclear factor erythroid 2-related factor 2 (NRF2), and ferroptosis suppressor protein 1 (FSP1) pathways [52] (Fig. 1).

### Lipid metabolism

Increased lipid peroxidation is a distinctive feature, and lipid peroxide metabolism is critical in ferroptosis [53]. Lipidomic analysis revealed that PUFAs, such as arachidonic acid (AA) and adrenoyl (ADA) that are phosphatidylethanolamines (PEs), are prime targets for peroxidation [54]. Ferroptosis is likely activated by the peroxidation of membrane phospholipids to produce phospholipid hydroperoxides (PLOOH) and the breakdown of PLOOH to generate 4-hydroxynonenal or malondialdehyde. Lipid peroxidation products cause membrane instability and permeabilization, ultimately leading to cell death. Lipid peroxidation occurs in both non-enzymatic and enzymatic ways. In non-enzymatic lipid peroxidation, PUFAs are ligated to coenzyme A (CoA) by operating acyl-CoA synthase long-chain family member 4 (ACSL4), which produces acyl-CoA. Following this, acyl-CoA could be re-esterified in phospholipids through lysophosphatidylcholine acyltransferases (LPCATs) to generate phospholipids [55]. Acyl-CoA synthetase ACSL4 and lysophosphatidylcholine acyltransferase 3 (LPCAT3) are critical drivers of ferroptosis [56, 57]. In enzymatic lipid peroxidation, lipoxygenase (LOX) catalyzes the oxidation of polyunsaturated fats into their corresponding hydroperoxides. Lipoxygenases are non-heme iron-containing dioxygenase enzymes [58]. Previous research has found that overexpression of LOX-5, LOX-12, and LOX-15 causes cells to become more vulnerable to ferroptosis. Additionally, inhibitors of LOX activity can protect cells from ferroptotic cell death [53].

### Iron metabolism

Iron is an indispensable trace element in the human body involved in various biological processes, including oxygen transport, ATP production, DNA biosynthesis, and cell proliferation [59]. However, excess iron can generate ROS and lead to oxidative damage. Therefore, iron homeostasis is tightly regulated to maintain a balance between iron absorption, utilization, and storage [60]. Iron regulatory protein (IRP) is a crucial regulator of endogenous iron homeostasis. IRP binds to specific RNA sequences called iron-responsive elements (IREs) located in the 5’- or 3’-untranslated regions of mRNAs encoding proteins involved in iron metabolism [61]. Cancer cells exhibit higher metabolic requirements than normal cells, and their survival and proliferation largely depend on iron [59]. Fe^3+^ in the peripheral circulation binds to transferrin and forms a complex; then, the complex is imported into the endosome through endocytosis mediated by transferrin receptor 1 (TFR1). Acidic conditions in endosomes cause the release of Fe^3+^ from transferrin, which is then converted to Fe^2+^ by the ferrireductase activity of the six-transmembrane epithelial antigen of the prostate 3 (STEAP3) [62]. Following this, Fe^2+^ is released from endosomes into a labile iron pool (LIP) under the control of divalent metal transporter 1 (DMT1, also known as SLC11A2 or Nramp2) [62, 63]. Increased intracellular LIP can produce free radicals (hydroxyl radicals) through the Fenton reaction and participate in the peroxidation of phospholipids to form PLOOH [64]. Ferritin is a protein complex composed of ferritin light chain (FTL) and ferritin heavy chain 1 (FTH1), which is capable of storing excess iron. The nuclear receptor co-activator 4 (NCOA4) directly binds to FTH1 to form the ferritin complex targeting lysosomes for “ferritinophagy”. Ferritinophagy causes increased intracellular iron levels and triggers ROS accumulation through the Fenton reaction, resulting in ferroptosis [65, 66]. Ferroportin (FPN1, also known as SLC40A1) is the only well-known iron exporter and plays a pivotal role in the export of Fe^2+^ from cells to the blood [67].

### Amino acid metabolism

Ferroptosis regulation is closely related to amino acid metabolism. Two key molecules in amino acid metabolism are GPX4 and system xc- [68]. GPX4 belongs to the glutathione peroxidase (GPX) family and contains selenocysteine in its catalytic center, which converts toxic lipid hydroperoxides (L-OOH) to a non-toxic form of lipid alcohol (L-OH) [69]. Glutathione (GSH) is a necessary substrate for the physiological functioning of GPX4. GSH is a critical antioxidant synthesized from glutamate, cysteine, and glycine; among these amino acids, cysteine is regarded as the rate-limiting precursor. System xc- is a sodium-independent antiporter composed of two subunits, i.e., the transporter subunit, solute carrier family 7 member 11 (SLC7A11), and the regulatory subunit, solute carrier family 3 member 2 (SLC3A2). This transporter system mediates the exchange of intracellular glutamate and extracellular cystine in a ratio of 1:1 [49, 70].

### p53

p53 is a human tumor suppressor gene activated in response to various forms of cellular stress, such as DNA damage, oxidative stress, and dysregulated metabolism [71]. P53 has a dual function in ferroptosis. p53 acts as a transcriptional repressor for SLC7A11, leading to reduced cellular cystine import, increased ROS accumulation, and ferroptosis induction [72]. Additionally, p53 can induce ferroptosis by increasing the expression of glutaminase 2 (GLS2) and spermidine/spermine N1 acetyltransferase 1 (SAT1) [73, 74]. On the other hand, p53 can suppress ferroptosis either by inhibiting dipeptidyl peptidase 4 (DPP4) or by inducing the activity of the cell cycle protein-dependent kinase inhibitor 1A (CDKN1A, also known as p21) [75, 76].

### Nuclear factor erythroid 2-related factor 2

NRF2 is a transcription factor crucial for regulating cellular antioxidant activity [77]. Under physiological circumstances, the expression of NRF2 is low, and KEAP1 closely controls its activity. However, in response to oxidative stress, NRF2 dissociates from KEAP1 and moves to the nucleus to activate the transcriptional gene, which functions as an antioxidant against oxidative stress and inhibits ferroptosis [78, 79].

### Ferroptosis suppressor protein 1

Ferroptosis suppressor protein (FSP1) is in the cytoplasmic membrane, and its anti-ferroptotic activity relies on N-myristoylation [80]. A reduced form of coenzyme Q (CoQ), known as ubiquinol, is maintained by FSP1 at the plasma membrane. Ubiquinol acts as an antioxidant by capturing lipid peroxyl radicals and preventing the diffusion of lipid peroxides, thereby inhibiting ferroptotic cell death [81].

## Ferroptosis in cancer

Growing studies have investigated the significance of ferroptosis in solid cancer models and affected patients. It has been reported that the expression levels of NRF2 and SLC7A11 are upregulated in esophageal squamous cell carcinoma tissues; NRF2 is associated with metastasis, TNM stage, and lymph node metastasis in patients with esophageal squamous cell carcinoma, and NRF2 overexpression leads to radioresistance development [82]. In gastric cancer patients, NRF2 upregulation is associated with inferior prognosis of patients [83]. Increased expression of NRF2 has been positively associated with lymph node metastasis and poor differentiation; also, high expression of NRF2 is associated with poor overall survival of patients with non-small cell lung cancer (NSCLC) [84]. Besides, increased expression of NRF2 is associated with poor recurrence and disease-free survival of breast cancer patients, and NRF2 knockdown decreases the proliferation of breast cancer cells [85].

It has been reported that SLC7A11 is upregulated in renal cell carcinoma, and its increased expression is associated with the inferior survival of affected patients; SLC7A11 silencing is associated with decreased migration, invasion, and proliferation of malignant cells [86]. In patients with epithelial ovarian cancers, high co-expression of SLC7A11 and GPX4 has been substantially associated with poor overall survival and progression-free survival of affected patients [87]. Increased expression of SLC7A11 has been associated with inferior 5-year survival of patients with NSCLC, and SLC7A11 silencing decreases the tumor volume in nude animal models [88]. In patients with oral cavity squamous cell carcinoma, SLC7A11 upregulation is considerably associated with perineural and lymphovascular invasion, recurrence-free survival, disease-specific survival, and overall survival [89]. Also, Sugano et al. have reported that SLC7A11 is associated with lymphatic vessel invasion, and its expression is associated with the relapse-free survival of patients with colorectal cancer [90]. Also, high expression of SLC7A11 is associated with worse overall survival of laryngeal squamous cell carcinoma patients [91].

GPX4 upregulation is significantly associated with poor disease-specific survival and overall survival of gastric cancer patients and its silencing decreases the cell proliferation of malignant cells [92]. Increased expression of GPX4 is associated with large invasion depth, advanced tumor stage, and high grade of tumors in patients with oral squamous cell carcinoma [93]. In thyroid cancer, low expression of GPX4 is associated with improved overall survival of affected patients, and GPX4 knockdown decreases the clonogenicity of thyroid cancer cells [94]. It has been reported that increased expression of GPX4 is associated with poor overall survival of NSCLC patients as well [95]. Also, GPX4 inhibition decreases the spheroid formation, cell viability, and migration of thyroid cancer cells [96]. Collectively, tumoral ferroptosis activation can lead to the suppression of oncogenesis in solid cancers. The following discusses the significance of ferroptosis in cancer chemotherapy, radiotherapy, and immunotherapy.

### Ferroptosis and chemotherapy

Chemoresistance is a considerable obstacle in cancer treatment. In this regard, ferroptosis dysregulation is involved in developing chemoresistance in various cancers. Cisplatin-resistant ovarian cancer cells stimulate autophagy, increase the autophagic degradation of FTH1, and suppress ferroptosis. Of interest, erastin-induced ferroptosis decreases the cell viability of cisplatin-resistant ovarian cancer cells [97]. In hepatocellular carcinoma, the knockout of GSTZ1, an inhibitor enzyme of NRF2, suppresses ferroptosis and increases sorafenib resistance [98]. In addition, etoposide-mediated metabolic reprogramming can increase lactate production in NSCLC cells, leading to GPX4 ubiquitination and ferroptosis resistance [99]. Ferroptosis stimulation via GPX4 knockdown or FIN56 administration can eliminate chemoresistant NSCLC cells [100]. In line with this, it has been shown that KLF11 suppresses GPX4 in lung adenocarcinoma, stimulates ferroptosis, and enhances their chemosensitivity to cisplatin [101]. In gastric cancer, the beta-catenin/TCF4 transcription complex promotes GPX4 expression and inhibits ferroptosis. Of interest, TCF4 overexpressed gastric cells are highly resistant to cisplatin [102].

In addition, chemotherapeutic agents can induce ferroptosis, and the dysregulation of this process can lead to chemoresistance [53]. Among these agents, temozolomide, a DNA alkylating agent, has shown effectiveness in treating glioblastoma multiforme [103]. Temozolomide induces ferroptosis through various pathways; Chen et al. have discovered that the androgen receptor induces resistance to temozolomide treatment in glioblastoma. They have reported that the curcumin analog induces the ubiquitination of the androgen receptor, which suppresses GPX4 and causes ferroptosis, thereby reversing temozolomide resistance in glioblastoma [104]. Furthermore, temozolomide exerts ferroptosis-inducing effects by activating NRF2/ATF4 at the mRNA and protein levels, thus increasing the expression of SLC7A11 and enhancing cystathionine gamma-lyase activity [105]. Moreover, temozolomide stimulates ferroptosis in glioblastoma cells by upregulating DMT1, a crucial protein in regulating iron homeostasis [106]. Therefore, the additive effect of ferroptosis activation on the anti-tumoral effect of chemotherapeutic agents on cell viability can be of interest and open a new era in cancer medicine. Recent findings have evaluated the efficacy of biomimetic/biocompatible formulations and pH-responsive liposomal nanoreactors for inducing ferroptosis in cancer models [107, 108].

### Ferroptosis and radiotherapy

Radiotherapy employs high-energy ionizing radiation to cause DNA double-strand breakage, which is a mainstream therapeutic approach for treating various cancers. Apart from the direct DNA damage, ionizing radiation can also trigger indirect cellular effects by radiolysis of cellular water and activating oxidases, producing ROS such as hydroxyl radicals and hydrogen peroxide, which can damage nucleic acids, proteins, and lipids. Recent findings have demonstrated that ionizing radiation induces ferroptosis in cancer cells [109]. Shibata et al. have reported that treatment with erastin, as a ferroptosis inducer, decreases the levels of glutathione and GPX4 and increases radiotherapy-mediated anti-tumoral effect in vivo [110]. It has been reported that the activating effect of radiotherapy on ferroptosis is dependent on p53; radiotherapy activates p53 and suppresses radiotherapy-mediated SLC7A11 expression, leading to glutathione synthesis inhibition and ferroptosis stimulation. Of interest, ferroptosis inducers that suppress SLC7A11 enhance the radiosensitivity of p53 mutated malignant cells [111]. Besides, ferroptosis activation via inhibiting system xc- or GPX4 inhibition can synergize the radiotherapy-mediated ferroptosis stimulation in cancers. In murine xenograft and patient-derived models, ferroptosis inducers increase the anti-tumoral effect of radiotherapy [112]. As mentioned above, KEAP1 closely controls the expression of NRF1, and KEAP1 mutation leads to continuous expression of NRF2. It has been reported that KEAP1 deletion increases the expression of SLC7A11, leading to radioresistance. However, the combined deletion of KEAP1 and SLC7A11 sensitized the malignant cells to radiotherapy [109]. Koppula et al. have reported that CoQ-FSP1 is another downstream effector from the KEAP1-NRF2 axis, and the FSP1 inhibition has been associated with increased radiotherapy response of KEAP1 deficient lung cancer cells via inducing ferroptosis [113]. In addition, estrogen receptor 1 can activate SLC7A11 expression, and estrogen receptor 1 knockdown increases ferroptosis in malignant cells; also, NEDD4L ubiquitinates SLC7A11 in estrogen receptor-positive breast cancer cells during radiation [114]. Consistent with this, Liu et al. have also reported that estrogen receptor 1 knockdown enhances radiation-induced ferroptosis in breast cancer cells [115].

Ataxia telangiectasia mutated (ATM) is an indispensable component of the DNA repair system activated following cytotoxic chemotherapy or radiotherapy. ATM mediates the downregulation of SLC7A11 after radiotherapy, resulting in ferroptosis; this anti-neoplastic effect of radiotherapy is enhanced by the combination of immune checkpoint inhibitors, e.g., anti-PD-L1 or anti-CTLA4 antibodies, in tumor models [116]. In line with this, it has been shown that radiotherapy can activate ATM, leading to the inhibition of SLC7A11, reduced cysteine uptake, ferroptosis activation, and tumor growth retardation [117]. Radiotherapy induces ACSL4 expression and triggers PUFA-PL peroxidation, subsequently causing ferroptosis. Of interest, suppression of SLC7A11 and GPX4 has been associated with sensitizing radioresistant malignant cells [109]. Irradiated cancer cells also release microparticles that can enhance radiotherapy by propagating signals that trigger ferroptosis or increase the expression of proteins involved in oxidative stress [118].

### Ferroptosis and immunotherapy

Cancer immunotherapy often encounters low response rates and treatment resistance in various tumors, which leads to unsatisfactory therapeutic outcomes [119]. Therefore, there is a need to increase the efficacy of cancer immunotherapy. In this regard, recent studies have shown that ferroptosis inhibits tumor growth and enhances the efficacy of immunotherapy through multiple signaling pathways, suggesting that ferroptosis induction may contribute to diminishing cancer immunotherapy resistance [120]. Yang et al. showed that the combination of GPX4 inhibition and anti-PD1 treatment enhanced tumor ferroptosis and augmented antitumor immune responses in triple-negative breast cancer [121]. Combined treatment of cyst(e)inase with PD-L1 blockade synergistically induces ferroptosis, suggesting that it could be a promising approach to enhance antitumor immunotherapy [122]. It has been reported that the tumor microenvironment of ACSL4^−/−^ animal models displays decreased infiltration of CD8^+^, IFNγ^+^CD8^+^, TNFα^+^CD8^+^, and CD4^+^ T cells compared to the tumor microenvironment of ACSL4^+/+^ animal models. Of interest, the combined treatment of PD-L1 blockade with the supplementation of arachidonic acid, as a ferroptosis-promoting agent, has enhanced the efficacy of PD-L1 blockade and increased the infiltration of TNFα^+^, IFNγ^+^, and granzyme B^+^CD8^+^ T-cells in animal models. Besides, arachidonic acid has no significant effect on the function or survival of T-cells in vitro [123]. Elevated GPX4 levels in Treg cells prevent lipid peroxidation and ferroptosis, while inhibiting GPX4 increases antitumor immunotherapy [124]. In addition, it has been indicated that CYP1B1 causes the metabolization of arachidonic acid to 20-HETE, leading to the PKC pathway-mediated FBXO10 upregulation. FBXO10 has been implicated in ACSL4 degradation, paving the way for ferroptosis resistance. The combined treatment of CYP1B1 inhibition increases the sensitivity of tumor cells to anti-PD-1 blockade in C57BL/6 J mice [125]. Aside from these pathways, ferroptotic stress increases the expression of PD-L1 and sensitizes head and neck cancer animal models to anti-PD-L1 treatment [126]. On the other hand, the activation of CD8^+^ T cells by anti-PD-L1 antibodies regulates ferroptosis in cancer cells. The IFN-γ released by CD8^+^ T cells in the tumor microenvironment downregulates the expression of two subunits of system xc-, consequently promoting the accumulation of lipid peroxidation and facilitating ferroptosis [122]. Cisplatin-induced ferroptosis is partly responsible for the synergistic anti-tumoral effect of the combination of cisplatin with anti-PD-1 antibody in animal models of lung cancer. Indeed, cisplatin-induced ferroptosis increases the infiltration of T-cells, N1-neutrophil polarization, and type 1 T helper differentiation [127].

However, the effect of ferroptosis stimulation should be distinctly studied on the malignant cells and tumor-infiltrating effector cells. Liu et al. have found that ferroptosis inhibition sensitized glioblastoma to anti-PD-1/anti-PD-L1 immunotherapy [128]. Within the tumor microenvironment, CD36-mediated fatty acid uptake by tumor-infiltrating CD8^+^ T cells leads to lipid peroxidation and ferroptosis. This results in reduced production of cytotoxic cytokines and impaired antitumor response. Therefore, blocking CD36 expression or using ferroptosis inhibitors combined with anti-PD-1 antibodies can enhance the antitumor effects of CD8^+^ T cells [129]. Conche et al. have reported that GPX4 deficient hepatocellular cells promote the immunosuppressive tumor microenvironment, which is characterized by the infiltration of CXCL10-related cytotoxic CD8^+^ T cells, tumoral PD-L1 upregulation, and HMGB1-mediated myeloid-derived suppressor cell (MDSC) infiltration; PD-1 blockade substantially increases the survival of GPX4 deficient animal models [130]. Kim et al. have shown that ferroptosis can limit human and mouse T-cell activity, and ferroptosis inhibition synergizes with immune checkpoint inhibitors and decreases cancer progression [131]. Therefore, the anti-tumoral effect of ferroptosis should be tailored to not influence the function or survival of effector immune cells and only enhance the ferroptosis of malignant cells. Overall, tumor ferroptosis modulation can be promising to increase the anti-tumoral effect of immunotherapy.

## Ferroptosis-regulating ceRNA networks

**Fig. 2:**
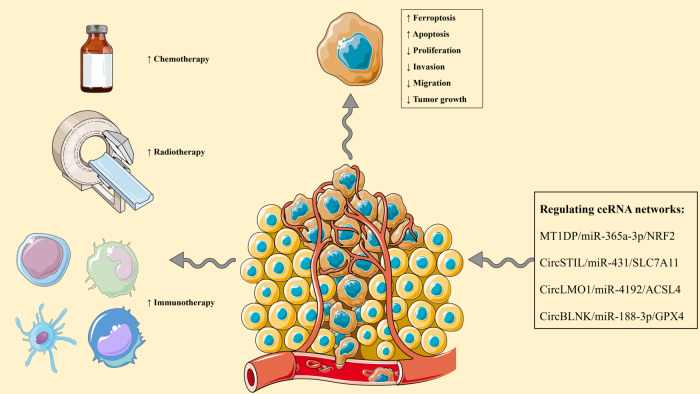
Restoring ferroptosis-regulating ceRNA networks increases cancer immunotherapy and chemoradiotherapy responses, increases ferroptosis, stimulates apoptosis, and decreases proliferation, migration, invasion, and tumor growth.

As discussed above, circRNA and lncRNA-associated ceRNA regulate a wide range of biological processes. Dysregulated ceRNA networks considerably dysregulate gene expression implicated in cancer development and progression. Given the significance of enhancing tumoral ferroptosis in improving the chemoradiotherapy and immunotherapy responses, identifying the dysregulated ferroptosis-related ceRNA networks in cancers is of substantial importance. In contrast to the pharmacological regulation of ferroptosis, gene therapy-mediated rectifying the dysregulated ferroptosis-related ceRNAs can regulate ferroptosis as well as other biological processes, like proliferation, migration, and apoptosis (Fig. 2). Because ceRNA networks regulate numerous genes both directly and indirectly. Therefore, the following discusses the available experimental evidence on the ferroptosis-related ceRNA networks in various cancers (Table 2).

**Table 2:** The identified ceRNA networks in regulating cancer ferroptosis.

	ceRNA	Effect on ferroptosis	Mechanism	Studied cancer	Reference
NRF2-regulating ceRNAs	MT1DP/miR-365a-3p/NRF2	Positive	MT1DP downregulates NRF2 via stabilizing miR-365a-3p.	Non-small cell lung cancer	[132]
	GMDS-AS1 and LINC01128/miR-6077/KEAP1/NRF2	Positive	GMDS-AS1 and LINC01128 sponges miR-6077 and liberate KEAP1 expression, leading to regulating NRF2 activity.	Lung adenocarcinoma	[133]
	CircOMA1/NRF2	Negative	CircOMA1 results in NRF2 upregulation.	Prolactinoma	[134]
ACSL4-regulating ceRNAs	CircLMO1/miR-4192/ACSL4	Positive	CircLMO1 sponges miR-4192, leading to ACSL4 upregulation.	Cervical cancer	[135]
	CircSCN8A/miR-1290/ACSL4	Positive	CircSCN8A sponges miR-1290, leading to ACSL4 upregulation.	Non-small cell lung cancer	[136]
GPX4-regulating ceRNAs	Linc00976/miR-3202/GPX4	Negative	Linc00976 sponges miR-3202, leading to GPX4 upregulation.	Cholangiocarcinoma	[137]
	HCG18/miR-450b-5p/GPX4	Negative	HCG18 sponges miR-450b-5p, leading to GPX4 upregulation.	Hepatocellular carcinoma	[138]
	CircBLNK/miR-188-3p/GPX4	Negative	CircBLNK sponges miR-188-3p, leading to GPX4 upregulation.	Osteosarcoma	[139]
	CircDTL/miR-1287-5p/GPX4	Negative	CircDTL sponges miR-1287-5p, leading to GPX4 upregulation.	Non-small cell lung cancer	[140]
	CircIL4R/miR-541-3p/GPX4	Negative	CircIL4R sponges miR-541-3p, leading to GPX4 upregulation.	Hepatocellular carcinoma	[141]
SLC7A11-regulating ceRNAs	CircP4HB/miR-1184/SLC7A11	Negative	CircP4HB sponges miR-1184, leading to SLC7A11 upregulation.	Lung adenocarcinoma	[143]
	FOXQ1/circ_0000643/miR-153/SLC7A11	Negative	FOXQ1 increases the circ_0000643 expression and circ_0000643 sponges miR-153, leading to SLC7A11 upregulation.	Breast cancer	[142]
	CircSnx12/miR-194-5p/SLC7A11	Negative	CircSnx12 sponges miR-194-5p, leading to SLC7A11 upregulation.	Ovarian cancer	[144]
	CircSTIL/miR-431/SLC7A11	Negative	CircSTIL sponges miR-431, leading to SLC7A11 upregulation.	Colorectal cancer	[145]
	Circ_0067934/miR-545-3p/SLC7A11	Negative	Circ_0067934 sponges miR-545-3p, leading to SLC7A11 upregulation.	Thyroid cancer	[146]
	CircEPSTI1/miR-375_miR409-3P_miR-515-5p/SLC7A11	Negative	CircEPSTI1 sponges miR-375, miR409-3P, and miR-515-5p, leading to SLC7A11 upregulation.	Cervical cancer	[147]
	BBOX1-AS1/miR-513a-3p/SLC7A11	Negative	BBOX1-AS1 sponges miR-513a-3p, leading to SLC7A11 upregulation.	Esophageal squamous cell cancer	[148]
	SNHG14/miR-206/SLC7A11	Negative	SNHG14 sponges miR-206, leading to SLC7A11 upregulation.	Osteosarcoma	[149]
	SLC16A1-AS1/miR-143-3p/SLC7A11	Negative	SLC16A1-AS1 sponges miR-143-3p, leading to SLC7A11 upregulation.	Renal cell carcinoma	[150]

*NRF2* nuclear factor erythroid 2-related factor 2, *KEAP1* Kelch-like ECH-associated protein 1, *ACSL4* acyl-CoA synthetase long-chain family member 4, GPX4 glutathione peroxidase 4, *SLC7A11* Solute carrier family 7 member 11, *FOXQ1* Forkhead box Q1.

### CeRNA networks regulating NRF2

MT1DP downregulates NRF2 via stabilizing miR-365a-3p and sensitized NSCLC cells to erastin-induced ferroptosis. The in vivo models have indicated that the delivery of MT1DP and erastin via folate-modified liposome decreases tumor growth; the in vitro results have shown that this co-delivery increases ferroptosis and decreases the migration, invasion, and proliferation of NSCLC cells [132]. It has been reported that GMDS-AS1 and LINC01128 lncRNAs increase the chemosensitivity of lung adenocarcinoma via sponging miR-6077 and the miR-6077/KEAP1 axis. As mentioned above, KEAP1 closely regulates NRF2, and the GMDS-AS1 and LINC01128/miR-6077/KEAP1 can control NRF2 and increases ferroptosis [133]. As an upregulated circRNA in chemoresistant prolactinoma tissues, circOMA1 increases tumor growth in vivo and in vitro and inhibits ferroptosis via facilitating GXP4 and NRF2 upregulation [134].

### CeRNA networks regulating ACSL4

As downregulated circRNA in cervical cancer tissues, circLMO1 overexpression decreases the proliferation and invasion and increases the ferroptosis of cervical cancer cells via the miR-4192/ACSL4 axis [135]. As downregulated circRNA in NSCLC tissues and cells, the decreased expression level of circSCN8A is associated with poor prognosis of affected patients. circSCN8A ectopic expression inhibits migration, proliferation, invasion, and tumor growth in animal models and increases ferroptosis via the circSCN8A/miR-1290/ACSL4 axis [136].

### CeRNA networks regulating GPX4

As an upregulated lncRNA in cholangiocarcinoma, linc00976 increased expression is associated with lymph node metastasis and inferior overall survival of affected patients. Besides stimulating ferroptosis, linc00976 knockdown decreases the proliferation and migration of cholangiocarcinoma cells via the linc00976/miR-3202/GPX4 axis [137]. It has been reported that HCG18 knockdown decreases cell proliferation, increases apoptosis, and enhances sorafenib resistance via activating ferroptosis in hepatocellular carcinoma; these effects are mediated via the HCG18/miR-450b-5p/GPX4 axis [138]. circBLNK is an upregulated circRNA in osteosarcoma tissues and cell lines that its knockdown results in decreased proliferation, increased apoptosis, and increased intracellular free iron and lipid ROS via the circBLNK/miR-188-3p/GPX4 axis [139]. It has been shown that circDTL is upregulated in NSCLC tissues and cell lines, and circDTL knockdown increases the apoptosis, ferroptosis, chemosensitivity, and in vivo tumor growth of NSCLC via the circDTL/miR-1287-5p/GPX4 axis [140]. As an overexpressed circRNA in hepatocellular carcinoma tissues and cells, circIL4R knockdown decreases proliferation and increases ferroptosis via the circIL4R/miR-541-3p/GPX4 [141].

### CeRNA networks regulating SLC7A11

Huang et al. have reported that FOXQ1 is an upregulated oncogenic transcription factor in breast cancer that binds to the promotor of circ_0000643 to increase its expression. FOXQ1 knockdown increases breast cancer ferroptosis and inhibits oncogenesis via the FOXQ1/circ_0000643/miR-153/SLC7A11 axis [142]. As an upregulated circRNA in lung adenocarcinoma, circP4HB ectopic expression increases tumor growth and suppresses ferroptosis via the circP4HB/miR-1184/SLC7A11 [143]. As an upregulated circRNA in chemoresistant ovarian cancer tissues compared to sensitive ones, circSnx12 knockdown enhances the chemosensitivity and increases apoptosis and ferroptosis of cisplatin chemoresistant ovarian cancer cells circSnx12/miR-194-5p/SLC7A11 axis [144]. CircSTIL is an increased circRNA in colorectal cancer, and its knockdown is associated with decreased proliferation and enhanced ferroptosis of colorectal cancer cells via the circSTIL/miR-431/SLC7A11 axis [145]. In thyroid cancer, circ_0067934 knockdown decreases proliferation and increases apoptosis and ferroptosis of malignant cells via the circ_0067934/miR-545-3p/SLC7A11 axis [146]. CircEPSTI1 is upregulated in cervical cancer cells, and its knockdown decreases tumor growth in vivo and in vitro and activates ferroptosis via the circEPSTI1/miR-375_miR409-3P_miR-515-5p/SLC7A11 pathway [147]. BBOX1-AS1 is an upregulated lncRNA in esophageal squamous cell cancer tissues, and its increased expression is associated with poor overall survival, lymph node metastasis, increased tumor size, and TNM stage in affected patients; BBOX1-AS1 knockdown results in decreased proliferation, invasion, migration, and increased apoptosis and ferroptosis via the BBOX1-AS1/miR-513a-3p/SLC7A11 axis [148]. In nutlin-3a-resistant SJSA1 cells, lncRNA-SNHG14 knockdown enhances the sensitivity of tumoral cells and activates the ferroptosis via the SNHG14/miR-206/SLC7A11 [149]. SLC16A1-AS1 is an upregulated lncRNA in renal cancer tissues, and its increased expression is associated with poor overall survival of patients; SLC16A1-AS1 knockdown suppresses the cell viability, migration, and invasion and enhances ferroptosis via the SLC16A1-AS1/miR-143-3p/SLC7A11 axis [150].

## Future perspective and concluding remark

Ferroptosis is a newly identified regulated cell death; this metabolically-driven iron-related cell death depends on polyunsaturated fatty acyl peroxidation. Growing studies have shown that activating tumoral ferroptosis inhibits tumor development in pre-clinical models. Besides, the genes regulating ferroptosis have significant prognostic values for affected patients. Further studies have highlighted that tumoral cell ferroptosis activation displays promising results in increasing the efficacy of cancer immunotherapy and chemoradiotherapy. Therefore, activating ferroptosis in malignant cells can be promising in treating solid cancers.

The identification of the hidden language between major ncRNAs has provided valuable information about the complex interaction of RNAs in oncogenesis. The circRNA and lncRNA-mediated ceRNA networks can regulate various signaling pathways. In this regard, studies have shown that ceRNA networks are highly dysregulated in cancer development and progression, leading to oncogenes upregulation and tumor-suppressive genes downregulation. Recent studies have highlighted the significance of ceRNAs in regulating cancer ferroptosis and identified ferroptosis-regulating ceRNA networks in cancers. Since lncRNA, circRNA, and miR dramatically regulate the expression of a wide range of genes and pathways, restoring dysregulated ferroptosis-regulating ceRNA networks can provide ample opportunities to not only regulate ferroptosis and benefit from the anti-tumoral effects of ferroptosis regulation in cancer treatment, it can regulate other dysregulated pathways, like the pathways implicated in cancer migration, invasion, stemness, etc. In addition to the beneficial role of tumoral ferroptosis activation on cancer immunotherapy and chemoradiotherapy, the broad range of targets of ferroptosis-regulating ceRNA networks can be excellent targets for cancer treatment. The present study provided the available evidence on the ferroptosis-regulating ceRNA networks in different solid cancers and displays their potential for cancer treatment.
